# Coexisting EGFR and TP53 Mutations in Lung Adenocarcinoma Patients Are Associated With COMP and ITGB8 Upregulation and Poor Prognosis

**DOI:** 10.3389/fmolb.2020.00030

**Published:** 2020-02-27

**Authors:** Chang Zheng, Xuelian Li, Yangwu Ren, Zhihua Yin, Baosen Zhou

**Affiliations:** ^1^Department of Clinical Epidemiology, First Affiliated Hospital of China Medical University, Shenyang, China; ^2^Department of Epidemiology, School of Public Health, China Medical University, Shenyang, China

**Keywords:** co-mutation, EGFR^L858R^, TP53, gene interaction, COMP, ITGB8

## Abstract

The heterogeneity of lung adenocarcinoma is driven by key mutations in oncogenes. To determine the gene expression, single nucleotide polymorphisms, and co-mutations participating in the initiation and progression of lung adenocarcinoma, we comprehensively analyzed the data of 491 patients from The Cancer Genome Atlas. Using log-rank and Kruskal–Wallis analysis, Oncoprint, Kaplan–Meier survival plots, and a nomogram, we found that EGFR^L858R^ with co-mutation TP53 was significant prognostic determinant versus that with co-wild TP53 (hazard ratio, 2.77, *P* = 0.012). Further gene co-expression network and functional enrichment analysis indicated that co-mutation of EGFR^L858R^/TP53 increases the expression of COMP and ITGB8, which are involved in extracellular matrix organization and cell surface receptor signaling pathways, thus contributing to poor prognosis in lung adenocarcinoma. Validation was performed using three GEO profiles along with colony formation and CCK-8 assays for proliferation, transwell and wound-healing for migration in transfected H1299 and A549 cell lines. To the best of our knowledge, these results are the first to indicate that patients harboring the co-mutation of EGFR^L858R^/TP53 show increased expression of COMP and ITGB8, which participate in extracellular matrix dysfunction and can be used as prognostic biomarkers in patients with lung adenocarcinoma.

## Introduction

Lung cancer remains the leading cause of cancer-related mortality worldwide, accounting for approximately 1.76 million deaths each year, of which lung adenocarcinoma (AC) accounts for over 40% (Bray et al., 2018). Surgical resection is regarded as the standard treatment for stage I–IIIa non-small-cell lung cancer (NSCLC), and the 5-year survival rate of early and locally advanced stages is 92 and 26%, respectively (Goldstraw et al., 2016). Since most lung AC patients present with locally advanced or metastatic cancer at initial diagnosis, their total 5-year survival is only about 15% (Imielinski et al., 2012). However, up to 50% patients encounter relapse after surgery, indicating that residual cancer cells could not be detected by the current techniques (Tomida et al., 2009). Lung cancer treatment based only on various clinical and pathological parameters is insufficient, and increasing researches have focused on identifying the genetic alterations driving cancer initiation and progression, which could have clinical benefits for treatment options and the lives of patients.

Use of novel sequencing technologies and bioinformatics has identified actionable biomarkers that drive tumor progression and oncogenes, allowing the development of appropriate targeted therapies. Almost 60% of NSCLC patients harbor at least one significantly mutated gene (SMG), and about half of these mutations can be intervened using targeted therapy (Zhou et al., 2011). In NSCLC, several SMGs such as epidermal growth factor receptor (EGFR), anaplastic lymphoma kinase (ALK), and other less established oncoproteins have been verified to serve as targets in personalized treatment (Tan et al., 2015). Moreover, mutations driving oncogenes demonstrate biological overlap to form combination mutations (Kris et al., 2014). Even though molecular diagnostics and targeted therapies have improved the survival status for patients with SMG, adaptive and therapeutic resistance is usually observed (Imielinski et al., 2012). These resistance mechanisms might occur through parallel and/or as collateral of activated signaling pathways and driver genes, thus developing off-target resistance. Furthermore, investigation on combined mutation of SMGs and the parallel or downstream signaling pathways that lead to off-target resistance can provide new effective therapeutic strategies.

In this study, we comprehensively analyzed the incorporated genetic polymorphism data, mutation frequency data from exome sequencing, and gene expression data from RNA sequencing (RNA-seq) of lung AC in The Cancer Genome Atlas (TCGA) Project to determine the gene expression, single nucleotide polymorphism (SNP), and co-mutation participating in the initiation and progression of lung AC to examine suitable options for a novel targeted therapy.

## Materials and Methods

### Data Set Retrieval and Preparation

#### Somatic Mutation and Clinical Data

Next generation sequencing data (exome sequencing and RNA-seq data) and clinical sample details of lung AC were downloaded from The Cancer Genome Atlas (TCGA) project. In total, 569 Mutation Annotation Format (MAF) files with somatic mutations and SNP data from simple nucleotide variation were identified using VarScan2 algorithm with the default parameters. The clinicopathological characteristics included age (>65, ≤65 years), gender, clinical stage, living status, and overall survival (OS) time. The samples without OS information were filtered out and those with survival longer than 1825 days were considered as living.

#### Processing of RNA-seq Data

The preprocessed RNA-seq data (level 3) from 515 cases were generated using the Illumina HiSeq 2000 RNA Sequencing Version 2 platform. The Human Gene Sets GTF annotation file of Human Genome version GRCh38.p12, downloaded from the Ensembl database was used as the reference gene transcript set. The RNA-seq data were normalized using the edgeR package (Robinson et al., 2010) of the R statistical software environment^1^, which performs false discovery rate (FDR) analyses.

### Statistical Analysis Pipeline

In total, 491 tumor samples overlapping in mutation, SNPs, RNA-seq, and clinical data sets were combined to study the effect of somatic mutations and SNP-associated RNA expression on the survival of lung AC patients. The combination and preparation of all datasets was conducted using Perl and R statistical environment. The workflow of the analysis is listed as follows, and is shown in Supplementary Figure S1.

The first step was to identify the association between common SNPs alteration (alternated in more than 20 samples) and those affecting gene expression or OS. Some SNPs were merged into the dominate model (DOM) because their mutated genotypes were rare. We applied the expression quantitative trait loci (eQTL) association to evaluate the potential effect of SNPs on gene expression using the Kruskal–Wallis test, visualized with ggplot2 R package in a violin plot (Hintze and Nelson, 1998). We also examined the correlation between SNPs alternation and OS using Cox proportional hazards regression (adjusted by gender, age, and clinical stage) and log-rank test, and drew Kaplan–Meier (K-M) survival plots using the survival package.

The second step was to identify and visualize the most common somatic mutations in AC patients using Oncoprint, and then detecting the highest frequency of a mutant gene co-mutated with a significantly functional SNP mutant. We evaluated the prognostic and predictive value of TP53 wild-type (WT) or mutation-type (MUT) combined with genotype rs121434568 (TT/TG) in EGFR (EGFR^L858R^). Mutation status was grouped into four classes: TP53-WT/EGFR^L858R^-TT, TP53-WT/EGFR^L858R^-TG, TP53-MUT/EGFR^L858R^-TT, and TP53-MUT/EGFR^L858R^-TG.

In the last step, we detected the differentially expressed genes (DIFF-genes) in the transcriptome between EGFR^L858R^ genotypes and TP53 mutation status using edgeR package. We only selected genes with raw counts > 10 for the analysis. The cutoff was set at an absolute value of log2 fold change ≥ 1(| log2FC| ≥ 1) and an FDR-value ≤ 0.05. DIFF-genes were visualized as a volcano plot using ggplot2 R package.

### Predictive Nomogram Construction and Validation

A multivariate Cox proportional hazards regression model was developed to predict the risk score of independent LUAD prognosis-related clinical factors and EGFR^L858R^/TP53 mutation. The nomogram was further built using the rms package based on Cox regression analysis to visualize the total scores and the probability of 1- and 3-year OS (Iasonos et al., 2008). The concordance index (C-index) was calculated to assess the discrimination of the nomogram. The nomogram prediction probability was determined using a calibration curve compared with the observed rates.

### Biological Implication of EGFRL858R/TP53 Mutation

To determine the functional synergistic relationship between genes and mutation status, we first constructed a co-expressed gene module. The relationship with EGFR^L858R^/TP53 mutation in lung AC patients was detected using the “WGCNA” (weighted gene co-expression network analysis) package in R (Langfelder and Horvath, 2008). We then selected a list of significantly associated genes, which were most correlated with the mutation, and uploaded them to the ClueGO application (Bindea et al., 2009; version 2019.02) of Cytoscape software to calculate the gene ontology biological process (GO-bp) enrichment with a strict cut-off of FDR < 0.05.

### Validation of the Microarray Data Set

Gene Expression Omnibus (GEO) datasets were considered eligible for evaluating gene integration according to the following criteria: (1) Studies harboring EGFR^L858R^ mutation with three more samples. (2) Studies with information on the technology and platform utilized. (3) Studies containing suitable normal groups as the control. GSE17373, GSE57422, and GSE11729 were selected based on these criteria. Details of each sample including annotation profiles were also downloaded from the GEO repository. Correlation of gene expression levels was performed using Pearson correlation coefficient analysis, as shown by R square (R^2^) in Prism 8.0 (GraphPad Software, San Diego, CA, United States).

### Cell Culture and Transient Transfections

A549 and H1299 lung adenocarcinoma cell lines were routinely cultured in RPMI 1640 medium (GIBCO Thermo Fisher Scientific, Waltham, MA, United States) containing 10% fetal bovine serum (FBS, Israel), 100 units/mL penicillin, and 100 mg/mL streptomycin (Solarbio, Beijing, China) in an incubator at 37°C with 5% CO_2_. In this study, we used A549 as EGFR while TP53 co-wild type and H1299 as the TP53 mutant type. The plasmid containing EGFR^L858R^ or negative control (NC) were purchased from GenePharma, Co., Ltd. (Shanghai, China), and were transiently transfected using jetPRIME Transfection Reagent (Polyplus Transfection, Illkirch, France) according to the manufacturer’s protocol, as described previously (Zheng et al., 2018). Cells were classified into three groups: (a) co-wild EGFR^WT^/TP53^WT^ (A549 transfected with NC), (b) co-mutant EGFR^L858R^/TP53^MUT^ (H1299 transfected with EGFR^L858R^), and (c) TP53^MUT^ control (H1299 transfected with NC). After transfection, cells were incubated for 48 h and then collected to assess the specific transfection of EGFR^L858R^ expression using qRT-PCR analysis. Transfection cells were retained for later use in the functional assays.

### RNA Isolation and Real-Time PCR Detection

Total RNA was extracted using an RNA isolation plus kit (Takara, Dalian, China) according to the manufacturer’s protocol. RNA was reverse-transcribed to cDNA using Prime Script RT reagent Kits (Takara), followed by amplification using the SYBR Green Master Mixture reagent (Takara) on the ABI 7500-Fast Real-Time PCR system (Applied Biosystems, Woburn, MA, United States). The cDNA amplification and cycling conditions were used as described previously (Zheng et al., 2018). The fold change in relative expression level was calculated using the 2^–ΔΔCt^ method, with GAPDH as the internal reference. The primers used are listed in Supplementary Table S2.

### Functional Assays

Cell colony formation potential was determined by the colony formation assay following the manufacturer’s protocol at 10 days after treatment. Transfected A549 and H1299 cells (5000 per well) were seeded in a six-well plate and the resulting colonies at 10 days were fixed. The number of colonies was then counted and images were taken.

Cell proliferation was determined using the cell counting kit-8 (CCK-8) assay (Promega, Beijing, China) following the manufacturer’s protocol. Transfected cells were plated in 96-well plates (3000 per well); at every 24 h 10 μL of CCK-8 solution was added to the respective wells and incubated for 90 min. Optical density (OD) values were measured at 450 nm.

Cell migration and invasion assays were conducted with 8 μmm-pore size chamber inserts in a 24-well culture plate (Corning, NY, United States). Transfected cells, 25 × 10^3^ in 250 μL were resuspended in serum free medium in the upper chamber per well for migration assays respectively. The lower chamber was filled completely with 700 μL medium containing 10% FBS as a chemoattractant. After 48 h of incubation at 37°C, cells that did not migrate from the upper chamber were removed using cotton swabs, while, those that migrated through the membrane were fixed in 4% paraformaldehyde, stained with 40, 6-diamidino- 2-phenylindole for 20 min, and washed three times with PBS. Cells that migrated into, or which invaded the lower chamber, were counted using an inverted microscope under x20 magnification. The assays were performed in triplicate, with data presented as the average number of invading cells.

Wound-healing assays were performed using the transfected A549 and H1299 cells seeded on 6-well plates. Cells were grown to confluent and pretreated with an artificial “wound” that was created by scratching with a sterile 200 μL pipette tip. Six microscopic fields of cell migration into the gap were photographed under microscopy (× 10) at 0 and 24 h post-wounding. The rate of gap closure was measured and calculated as wound-healing in μm/hour.

Data obtained from the cell functional assays were expressed as the mean ± SEM. Each experiment was independently conducted three times. The statistical differences between two groups were analyzed using the Student’s *t*-test while differences among more than two groups were analyzed by one-way ANOVA using GraphPad Prism 8, a GraphPad Software program (San Diego, CA, United States).

## Results

### The Three Most Common SNPs in the Cancer Genome Atlas Cohort

In this analysis, we mainly determined the influence of the three most common SNPs alleles on gene expression and prognosis in lung AC.

The characteristics of 491 lung AC patients in the present study from TCGA cohort are displayed in Supplementary Table S1, including the patients’ gender, age at diagnosis, and cancer clinical stage. MAF profiles were evaluated and three common SNPs were detected as follows: 64 and 60 tumors harbored the rs121913529 and rs121913530 mutation in KRAS, respectively, and 21 tumors harbored the rs121434568 mutation in EGFR. The wild genotype of rs121913529 in KRAS was CC, the other mutations were considered as DOM because of few genotypes in each polymorphism (CA 27, CG 13, CT 15, GG 2, TT 1). The same situation was also observed with rs121913530 of KRAS where the DOM model contained the genotypes of CA 49, CT 2, AA 2, and TT 1 samples. Finally, 21 samples carried the TG genotype of EGFR^L858R^, mutated from the TT genotype.

**FIGURE 1:**
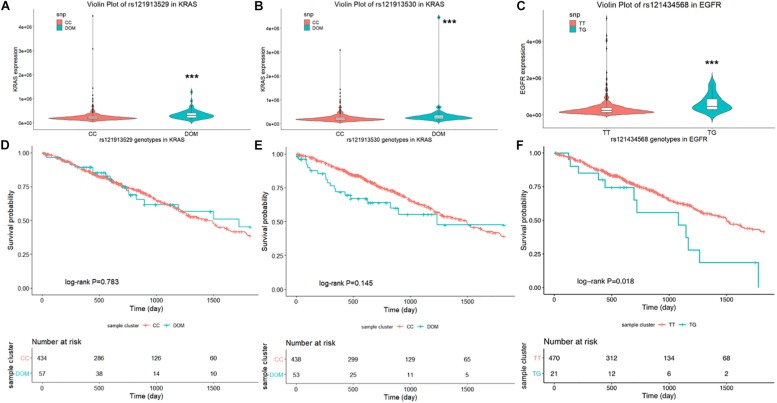
Violin plot displays the distribution of mRNA expression levels in each genotype. In the lung AC SNPs rs121913529 **(A)** and rs121913530 **(B)** in KRAS, rs121434568 in EGFR **(C)**, the Y-axis represents the relative expression level of harboring genes and the plot with error bars representing the 25 and 75% quantiles. Kaplan–Meier survival plot for SNPs: **(D)** rs121913529, **(E)** rs121913530 and **(F)** rs121434568. The unadjusted Kaplan–Meier curve was on the top and the number of events were showed in the risk table below. ***P < 0.001.

First, the violin plot in Figure 1 compares the level of mRNA expression between the wild- and mutant-type of rs121913529 and rs121913530 in KRAS, and rs121434568 in EGFR, respectively. The dominant model (colored in turquoise) of rs121913529 (Figure 1A) and rs121913530 (Figure 1B) in KRAS was highly expressed compared to the CC genotype (colored in orange). A similar trend was found in the EGFR^L858R^ (Figure 1C). The results of eQTL analysis indicated that these three mutation SNPs showed significantly higher expression than the wild-genotypes (*P* > 0.001).

Secondly, we examined the influence of these three SNPs on OS in lung AC patients. Compared to the reference wild-genotypes, both rs121913529 and rs121913530 DOM in KRAS were not related to prognosis, which can be observed when testing Cox proportional regression or plotting the K-M curve. Only rs121434568 genotype in EGFR showed a significant correlation with OS (TT vs. TG, HR, 1.99 [95%CI, 1.11–3.59]; *P*, 0.022; Table 1), adjusted by gender, age, and cancer stage. Univariate log-rank analysis was followed by the multivariate Cox proportional regression model to test the survival probability between EGFR^L858R^ TT- (470 samples) and TG- (21 samples) polymorphic alleles (Figures 1D–F). The K–M survival plot showed that patients harboring EGFR^L858R^ had a significantly shorter OS compared to those with EGFR^WT^.

**TABLE 1:** Cox regression analysis in most frequent SNP on overall survival in lung AC.

Gene (site)	SNP (nucleotide)	Codon	Amino acid	Median, days	No.(death)	HR^a^(95%CI)	*P*^a^
KRAS (exon 2)	rs121913529(CC)	GGT	Gly (12)	1454	434(150)	1.00	
	Dom model	GTT/GCT/GAT	Val/Ala/Asp	1725	57(19)	1.28(0.95–1.74)	0.109
	rs121913530(CC)	GGT	Gly (12)	1498	438(150)	1.00	
	Dom model	TGT/AGT	Cys/Ser	1235	53(19)	1.30(0.80–2.10)	0.292
EGFR (exon 21)	rs121434568(TT)	CTG	Leu (858)	1501	470(157)	1.00	
	rs121434568(TG)	CGG	Arg	1081	21(9)	1.99(1.11–3.59)	0.022

*HR^*a*^(95%CI), Hazard ratios, 95% confidence intervals, adjusted by age, gender, and cancer stage; *P*^*a*^, *P*-values, adjusted by age, gender, and cancer stage.*

### TP53 Mutation Identified in the EGFR L858R Subset

**FIGURE 2:**
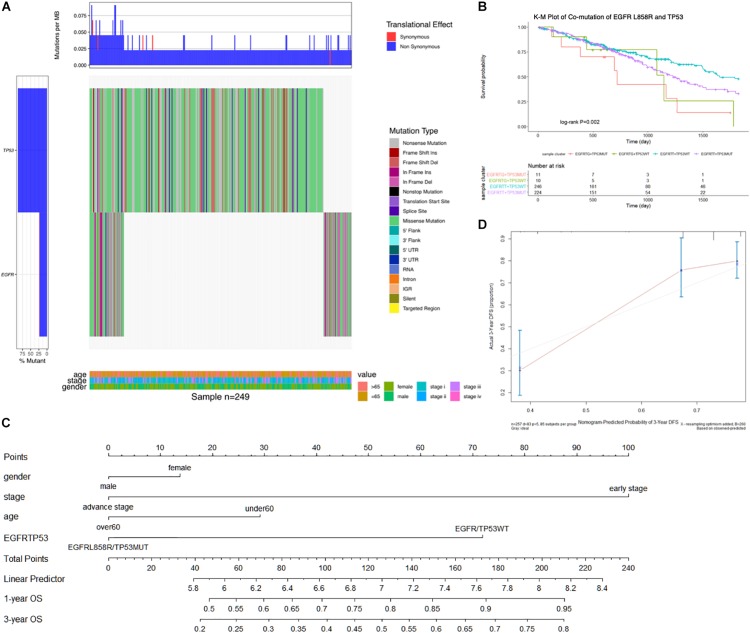
**(A)** The oncoprint waterfall plot of predictive combination effect of TP53 and EGFR mutation in lung AC. The mutation was colored as the legend in the right of plot, the left barplot indicated the probability of genetic mutation in each patient, while the upper barplot showed genetic mutation per gene. In the bottom was annotated by age, gender, and cancer stage. **(B)** Kaplan–Meier curve plot presented association between clinical outcomes and combination of EGFR^L858R^ and TP53 mutation. **(C)** Nomogram was constructed to combine signature with clinical features and EGFR^L858R^/TP53 mutation status by summing the scores. **(D)** Calibration plot indicated the nomogram had a predictive power for overall survival. EGFRTG + TP53MUT, EGFR^L858R^ with TP53^MUT^ status; EGFRTG + TP53WT, EGFR^L858R^ with TP53^WT^ status; EGFRTT + TP53WT, EGFR^WT^ with TP53^WT^ status; EGFRTT + TP53MUT, EGFR^WT^ with TP53^MUT^ status. The unadjusted Kaplan–Meier curve was on the top and the number of events were showed in the risk table below.

In a total of 491 samples, the most common somatic mutation was located in TP53, observed in 272 (55.4%) samples with variance types (mostly missense mutation). TP53 mutations are frequently reported to play multiple roles in lung cancer; hence, we combined TP53 with EGFR mutation to detect their effects on survival benefit. We selected 249 samples with common variants in TP53 and EGFR highlighted by an Oncoprint waterfall plot (Figure 2A), for which the mutation status and types are showed in the middle of the plot, annotated by the legend on the right. Age, clinical stage, and gender were listed at the bottom of the Oncoprint, showing that there was no correlation between the specific gene mutations and clinical features.

To test the association between co-mutation in TP53 and EGFR^L858R^ polymorphic alleles and OS, Cox proportional regression analysis was performed, and was adjusted by age, gender, and cancer stage. The most significant association with OS was observed in EGFR^L858R^/TP53^MUT^ type compared with the reference wild-type (HR, 2.77 [95%CI, 1.26–6.10]; *P*, 0.012; Table 2). There was a significant association between the EGFR^L858R^/TP53^MUT^ group (11 patients) and co-wild group (246 patients) in the K-M curve plot (*P*, 0.018; Figure 2B). Furthermore, a nomogram was constructed to predict the 1- and 3-year survival probability in patients that integrated clinicopathological features with the co-mutation of EGFR^L858R^/TP53 signature (Figure 2C). The nomogram of Cox multivariable model regression showed the risk score of the gender, clinical stage, age, and EGFR^L858R^/TP53 mutation status. Calibration plot was closely identical to diagonal line, showing good calibration of the 3-year survival probabilities predicted by the nomogram (Figure 2D). The concordance index (c-index) was 0.712 in this nomogram model, which indicated the predictive ability for the 3-year OS rates of LUAD patients harboring the EGFR^L858R^/TP53 mutation.

**TABLE 2:** Cox proportional regression analysis in co-mutation of EGFR^L858R^/TP53 on overall survival in LUAD.

Co-mutation type	Median, days	n.(event)	HR^a^ (95%CI)	*P*^a^
EGFR^WT^/TP53^WT^	1653	246(76)	1.00	
EGFR^WT^/TP53^MUT^	1229	224(81)	1.26(0.92–1.74)	0.156
EGFR^L858R^/TP53^WT^	1147	10(5)	1.77(0.71–4.40)	0.217
EGFR^L858R^/TP53^MUT^	719	11(7)	2.77(1.26–6.10)	0.012

*HR^*a*^(95%CI), Hazard ratios, 95% confidence intervals, adjusted by age, gender, and cancer stage; *P*^*a*^, *P*-values, adjusted by age, gender, and cancer stage; EGFR^*WT/L*858*R*^, rs1214568 genotype TT/TG in EGRF; TP53^*WT/MUT*^, TP53 wild/mutant type.*

### Significant Related Gene Network in Co-mutation of EGFR^L858R^/TP53

**FIGURE 3:**
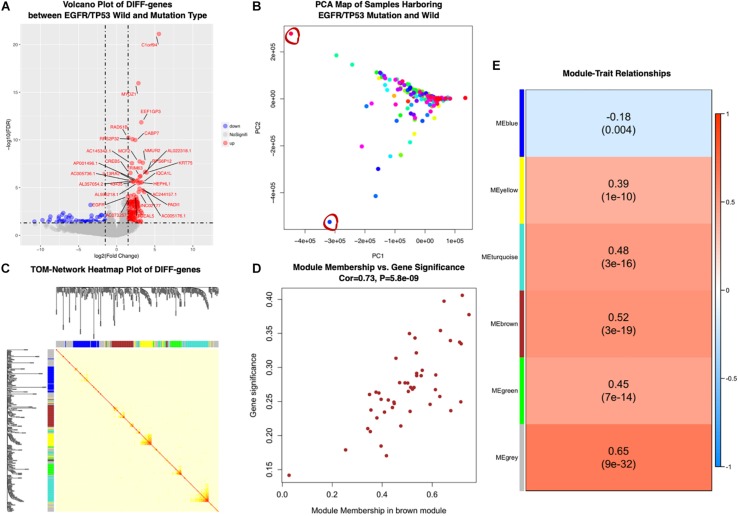
**(A)** Differentially expressed genes in EGFR^L858R^/TP53^MUT^ versus EGFR^WT^/TP53^WT^ samples. Volcano plots showed fold change and *P*-values of differentially expressed genes. Blue nodes presented significantly down-regulated, and red nodes were up-regulated expressed genes. Gray nodes were not differentially expressed. Genes with | log2FC| ≥ 1.5 expression were annotated. **(B)** Biplot principal component analysis (PCA) of EGFR^L858R^/TP53^MUT^ and EGFR^WT^/TP53^WT^ subgroup based on differential expressed genes levels. The outlier samples were significant with red circles. **(C)** Topological Overlap Matrix (TOM) of the differential expressed genes related to EGFR^L858R^/TP53^MUT^, visualized in heatmap (middle) and dendrogram to diagnose modules (along the left side and the top). **(D)** Scatter plot of module membership related to gene significant correlation in brown module. **(E)** Heatmap presents the correlation between module eigengenes and EGFR/TP53 mutant status in lung AC. DIFF-gene, differentially expressed genes; NoSignifi, no significant value; PCA, principal component analysis; cor, correlation rate.

According to the former Cox proportional regression analysis, we examined the significant DIFF-genes using TCGA transcriptome profiles between EGFR^L858R^/TP53^MUT^ (as the co-mutation group for 11 patients), which had poor prognosis, and EGFR^WT^/TP53^WT^ (as co-wild group for 246 patients). In total, 222 up- and 111 down-regulated expression genes were correlated with EGFR^L858R^/TP53^MUT^, as shown by the volcano plot in Figure 3A.

Then, the co-expression modules of these 333 genes most related to EGFR^L858R^/TP53^MUT^ were identified using WGCNA package. We first performed principal component analysis (PCA) to exclude two outlier samples (TCGA-55-7815-01 and TCGA-MP-A4TE-01, depicted as red circles in Figure 3B), and then examined the RNA expression pattern in 244 samples with both wild- and 11 mutated-type tumors. Five modules (blue, yellow, turquoise, brown, and green) were identified, and β = 5, was set as the lowest power for scale-free TOM construction (Figure 3C). Module-trait relationships were calculated by correlating MEs with mutation as shown in Figure 3E. The genes in brown modules were highly related with EGFR^L858R^/TP53^MUT^ carrying patients (correlation rate = 0.52, *P* < 0.001). Among the brown module genes, 43 were identified based on highly corresponding gene significance and MEs (correlated rate = 0.73), showing a significant relationship with mutation in lung AC (Figure 3D).

### Co-mutation of EGFR^L858R^/TP53^MUT^ Related to Cell Surface Receptors

**FIGURE 4:**
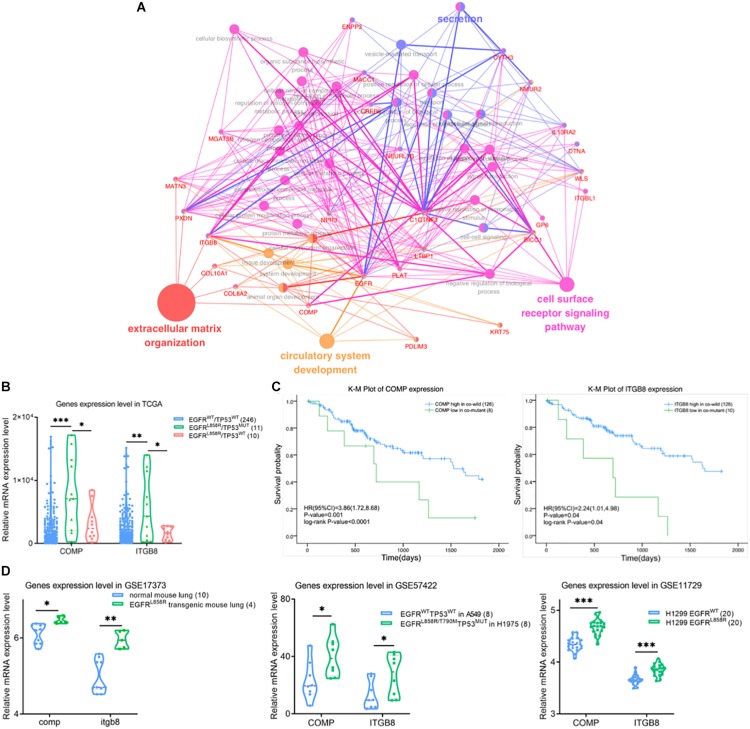
**(A)** Gene ontology biological process enrichment network analysis using ClueGO for differentially expressed genes in brown module. The color of nodes represents the gene enriched in corresponding GO-bp terms. **(B)** Violin plot of differential expression level of COMP and IGTB8 between co-wild EGFR/TP53, EGFR^L858R^/TP53^MUT^ and EGFR/TP53^MUT^ groups. Kaplan–Meier survival plot for COMP and IGTB8 **(C)** combined with mutation state of EGFR^L858R^ and TP53. The unadjusted Kaplan–Meier curve was on the top and the number of events were showed in the risk table below. **(D)** Correlations were detected between COMP or IGTB8 and EGFR expression level in GSE17373, GSE57422 and GSE11729 datasets, respectively. The sample were present in the figure legends. ^∗^*P* < 0.05, ^∗∗^*P* < 0.01, ^∗∗∗^*P* < 0.001. NC, negative control; WT, wild-type; MUT, mutant type.

We performed functional enrichment interaction analysis to explore GO-enriched categories in the genes of the brown module, which were most significantly related to EGFR^L858R^/TP53^MUT^. To assess the biological relevance of the brown module, 43 genes in the brown module were subjected to GO-bp functional enrichment analyses using ClueGO application in Cytoscape software (Figure 4A). The most frequent GO functions of proteins in the brown module were extracellular matrix (ECM) organization, cell surface receptor signaling pathway, and circulatory system development.

COMP and ITGB8 were found as core nodes of the cell surface ligand-receptor axis in ECM, but were also significantly upregulated in EGFR^L858R^/TP53^MUT^ tissues (11 samples) compared with either co-wild type (246 samples) or only EGFR^L858R^ mutant (10 samples) in Figure 4B. Based on the median relative expression level of COMP or ITGB8, patients were classified as high and low expression groups. A K-M curve drawn to compare the clinical outcomes between high and low expression levels of COMP or ITGB8, respectively, revealed that higher expression levels of ITGB8 or COMP affected the OS of patients harboring EGFR^L858R^/TP53^MUT^ compared with lower expression levels in co-wild EGFR/TP53 (Figure 4C). These data indicate that EGFR^L858R^/TP53^MUT^ could influence lung AC tumorigenesis in principle, through the COMP/ITGB8 axis as the cell surface ligand-receptor.

To evaluate the EGFR^L858R^/TP53^MUT^ status-regulated COMP and ITGB8 expression levels, samples in GSE17373, GSE57422, and GSE11729 were retrieved to analyze consistency with TCGA data profiles (Figure 4D). In the GSE17373 dataset, itgb8 expression level was significantly higher (1.2-fold change, *P*, 0.003) in EGFR^L858R^ transgenic mouse lung tissues compared with normal mouse, while comp showed a 1.06-fold change with a *P*-value of 0.01. In the GSE57422 dataset, a comparison of H1975 harboring EGFR^*L*858*R/T*790*M*^ and TP53^MUT^, with A549 harboring dual wild status EGFR and TP53, and showed consistent upregulated expression of COMP (2.08-fold change, *P*, 0.04) and ITGB8 (1.73-fold change, *P*, 0.03). Similar results were obtained from a comparison of H1299 transgenic with EGFR^L858R^ in GSE11729 (COMP, 1.08-fold change, *P* < 0.001; ITGB8, 1.05-fold change, *P* < 0.001).

**FIGURE 5:**
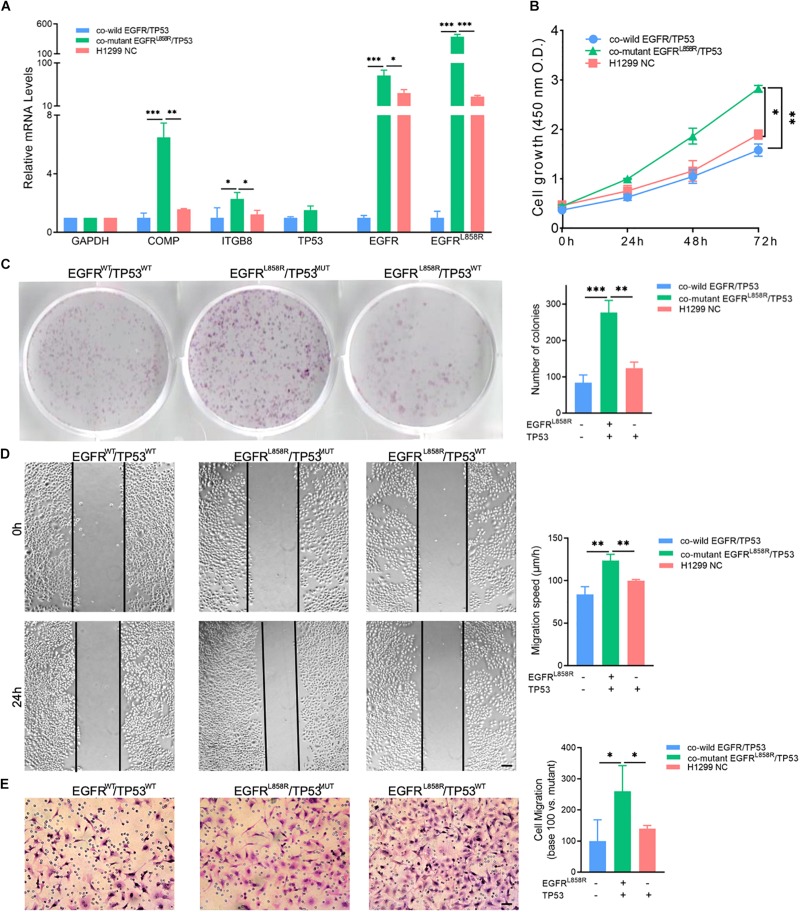
**(A)** Expression of COMP, ITGB8, TP53, EGFR, and EGFR^L858R^ in H1299 and A549 cells after transfection. **(B)** CCK-8 assay detected the proliferation by the value of OD_490_nm. **(C)** Representative cell colony formation assay and quantitative representation detected the proliferation of H1299 and A549 cells after transfection with EGFR^L858R^ or NC in A549 or H1299. **(D)** Representative images of initial and final wounds in A549 or H1299 cells after transfection with EGFR^L858R^ and negative control by wound-healing assay (Scale bar: 200 μm). **(E)** Representative transwell migration assays and quantitative representation after transfection with EGFRL858R or NC in A549 or H1299 cells (Scale bar: 100 μm). Assays were performed in triplicate. ^∗^*P* < 0.05, ^∗∗^*P* < 0.01, ^∗∗∗^*P* < 0.001. Error bars represent SEM. NC, negative control; WT, wild-type; MUT, mutant type.

Furthermore, we induced EGFR^L858R^ overexpression in H1299 cells by transfection and the NC in A549 and H1299 to detect the biological function of co-mutant EGFR^L858R^/TP53^MUT^. COMP and ITGB8 RNA expression levels were shown to be elevated in the co-mutant EGFR^L858R^/TP53^MUT^ with a corresponding fold change of 6.49 and 2.29, respectively, compared with the levels in the A549 NC group (Figure 5A). The CCK-8 (Figure 5B) and cell colony formation assays (Figure 5C) indicated that co-mutant EGFR^L858R^/TP53^MUT^ promoted cell proliferation ability compared to the co-wild A549 NC and TP53^MUT^ H1299 NC. Additionally, the role of the co-mutant EGFR^L858R^/TP53^MUT^ in LUAD cell migration was investigated using H1299 cells transfected with the EGFR^L858R^ overexpression plasmid. The results revealed that EGFR^L858R^/TP53^MUT^ resulted in increases migration abilities compared with A549 and H1299 cells in the NC group (Figures 5D,E). These results indicate that co-mutant EGFR^L858R^/TP53^MUT^ can promote the proliferation and metastasis of LUAD cells.

## Discussion

Genomic mutational heterogeneity in lung AC is related to cancer progression, drug resistance, and post-surgical relapse of localized tumors (Greaves and Maley, 2012). Subgroups of intratumor heterogeneity in lung AC could be classified by SMGs, and correlated with variance clinical outcomes and downstream signal pathways. In an earlier study of somatic mutation analysis in 188 lung AC patients, TP53, KRAS, STK11, and EGFR were the most frequent SMGs (Ding et al., 2008). There were 11 lung AC patients harboring the co-mutation of EGFR and TP53 among 34 EGFR mutations (Ding et al., 2008). Along with these mutations in EGFR coupled with TP53, patients in several other studies were found to harbor additional co-occurring driver mutation and burdens, which could have the capacity to affect clinical outcome by inflicting genomic instability (Cortot et al., 2014; Shepherd et al., 2017; VanderLaan et al., 2017).

Our results reported EGFR rs121434568, also known as EGFR^L858R^, as a functional SNP mutation by eQTL analysis. EGFR^L858R^ was correlated with poor clinical outcome in the TCGA cohort. We also found that neither KRAS rs121913529 nor rs121913530 genotypes were significant prognostic markers in lung AC, similar to a previous study (Shepherd et al., 2013). Patients harboring activated EGFR or KRAS mutation were mostly defined as 2 non-overlapping subgroups at the molecular level, showing large diversity in response to the same tyrosine kinase inhibitor (TKI) therapy (Chen et al., 2012). The kinase domain of EGFR received an objective response to targeted treatment with EGFR TKIs (Mok et al., 2009; Sangpheak et al., 2019). On the contrary, KRAS mutation was mostly resistant to TKI therapy, but might show sensitivity to a combination of selumetinib and chemotherapy (Soria et al., 2017). The mechanisms associated with the different responses to TKI therapy were hypothesized as EGFR mutations with variant downstream co-activated gene and pathway mutations (Lee et al., 2013; Eng et al., 2015; Gong et al., 2018). We next analyzed the mutation of EGFR combined with downstream TP53, and explored how this co-mutation influences prognosis.

TP53 is a common tumor suppressor mutation in NSCLC, constituting 46% mutations in lung AC of the TCGA cohort, consistent with a former study (Cancer Genome Atlas Research Network, 2014). Although TP53 was the most frequently mutated oncogene, the genetic changes in this gene were multiple and complex, and we did not analyze the function of single the TP53 genotype in the present study. Our results demonstrate that two SNP genotypes in KRAS showed no different influence on the patient OS, so we focused on alteration of TP53 followed by the EGFR^L858R^ mutation. The EGFR^L858R^/TP53^MUT^ acted as a stronger predictive prognostic factor and influenced the survival benefit, patients carrying EGFR^L858R^/TP53^MUT^ had significantly deteriorated OS, compared to EGFR^WT^/TP53^WT^ tumors. Interestingly, a single mutation in patients harboring TP53 or EGFR^L858R^ was not statistically significant in the prognosis compared with the EGFR^WT^/TP53^WT^ type. We could thus hypothesize that EGFR^L858R^ usually acted as a poor prognosis biomarker for NSCLC, and these negative results were caused by a combination of TP53 mutations.

Results from early reports underestimated the functions of this co-mutation in NSCLC, and emerging evidences suggest that EGFR mutation systematically accompanied the TP53 mutation in lung AC, thus affecting resistance to EGFR TKIs therapy and poor prognosis, which is consistent with our study. For example, Canale Matteo’s group analyzed the co-mutation of TP53 and EGFR, and showed worsened prognosis and reduced responsiveness to first-line TKIs (Canale et al., 2017). Catherine Labbé’s group found dual TP53/EGFR mutations in 41% of 105 NSCLC patients; especially, missense mutation of TP53 resulted in reduced response rates and shortened OS with TKI therapy (Labbé et al., 2017). The above studies endorse our hypothesis that co-mutation of TP53 and EGFR is a key to predict the prognosis of lung AC patients. According to our results, it is reasonable to assume that the various prognoses of patients and their response to TKIs therapy may depend, at least partially, on the co-mutation of TP53 and EGFR followed by cellular functions and pathways.

Next-generation sequencing platforms provide a powerful approach to explain the alteration of gene/pathways and to understand the biological basis of TP53 mutation, which impacted the survival of patients harboring the EGFR^L858R^-TG/TT genotypes. Firstly, the somatic mutations strongly influenced the process of transcription and gene expression level in cancers (Pleasance et al., 2010). According to the significance analysis, DIFF-genes correlated with patients harboring EGFR^L858R^/TP53^MUT^ were obtained.

Secondly, the key downstream or parallel pathways and processes lead to resistance against TKI therapy and poor clinical outcomes in patients (Sequist et al., 2007). The DIFF-genes were clustered by WGCNA into modules; EGFR^L858R^/TP53^MUT^ upregulated COMP and ITGB8 expression through various cellular signals, such as extracellular matrix (ECM) organization, and the cell surface receptor signaling pathway. Although little is known about EGFR^L858R^/TP53^MUT^, a previous study implied that tumors with overexpression of COMP and ITGB8 showed metastasis and invasion-related cancer. ITGB8 is a member of the integrin β-chain subfamily to form integrin complexes, thus mediating interactions between cell-cell and -ECM in cancers (Calderwood et al., 2013). The integrin complexes also functioned as signal transductor and regulated cell growth and motility (Calderwood et al., 2013). ITGB8 has been reported to be overexpressed in various cancers, especially in lung cancer and lung AD cell lines (Xu and Wu, 2012) and has recently been found to be related to gefitinib and cisplatin resistance in cancer (Cui et al., 2018). ITGB8 was also found to be amplified in the EGFR-mutated group of lung cancer (Blons et al., 2008). The integrin α1β1, similar to other integrins in the group of cell adhesion molecules, has been shown to interact with ANGPTL1 by inhibiting the JAK2/STAT3 signaling pathway (Yan et al., 2017). Recently, it was identified that EGFR^L858R^ mutation could repress the p53/p14 (Arf) pathway through STAT3/Bcl-2 signaling and counteract the pro-apoptotic function (Ozenne et al., 2013). Another study reinforced that Vps34 could regulate the EGFR/Arf pathway leading to apoptosis (Dayde et al., 2016). Considering these possibilities together, EGFR^L858R^/TP53^MUT^ associated poor prognosis of lung AC might occur through ITGB8 and its downstream signaling activation. COMP encodes cartilage oligomeric matrix protein, which is also an ECM protein that enhances the invasion of cancers through integrin binding (Posey et al., 2018). Taken together, COMP and ITGB8 play necessary roles in the integrity of ECM network. Currently, we found that activated EGFR^L858R^/TP53^MUT^ might promote COMP and ITGB8 expression that might be involved in ECM dysfunction during tumor development. However, mechanisms underlying the relationship between EGFR^L858R^/TP53^MUT^ and COMP/ITGB8 in lung cancer should be the focus of future studies.

Indeed, our results have the limitation of a small number of patients in the TCGA lung AC cohort. Considering this, we did not consider clinical stages as an individual confounding factor to influence OS. Further studies with a larger sample size of patients should be performed to validate our results with caution. However, rapid evolution of next-generation sequencing technology for gene mutations with liquid-based cytology and circulating free DNA has resulted in novel predictive diagnosis and targeted therapy (Couraud et al., 2014; Reynolds et al., 2017; Loong et al., 2018). Thus, co-mutation of TP53 and EGFR can be a strong predictive biomarker in prognostics and in determining personalized treatment strategies for patients with lung cancer.

## Conclusion

Taken together, both mutation in EGFR^L858R^ and co-mutation of EGFR^L858R^/TP53 act as a poor signal for the survival of lung AC patients. To the best of our knowledge, we report for the first time that tumors harboring co-mutation of EGFR^L858R^/TP53 may have poor prognosis owing to the upregulated COMP and ITGB8 expression in extracellular matrix and cell surface receptor signaling. High expression level of COMP and ITGB8 in EGFR^L858R^/TP53 mutant patients may thus be a novel option for TKI therapy selection. Further clinical trials are needed to evaluate the influence of this co-mutation on TKI therapies.

## Data Availability Statement

The TCGA datasets can be found in the (http://cancergenome.nih.gov) and GEO in (https://www.ncbi.nlm.nih.gov/gds) accession GSE17373, GSE57422, and GSE11729. The remaining data are available from the corresponding author upon request.

## Author Contributions

CZ and XL: designation, analysis, visualization, and original draft preparation. YR and ZY: methodology and software. BZ and XL: funding acquisition. All authors read and approved the final manuscript.

## Conflict of Interest

The authors declare that the research was conducted in the absence of any commercial or financial relationships that could be construed as a potential conflict of interest.

## Footnotes

1 http://www.r-project.org
