# Supplementary material for: Coexisting EGFR and TP53 Mutations in Lung Adenocarcinoma Patients Are Associated With COMP and ITGB8 Upregulation and Poor Prognosis
Source: Front Mol Biosci. 2020 Feb 27;7:30. doi: 10.3389/fmolb.2020.00030 (PMC7056714; doi:10.3389/fmolb.2020.00030)

[Table S1](http://clincancerres.aacrjournals.org/content/23/20/6078.long#T1). Displays the baseline characteristics of 491 patients in lung adenocarcinoma cohort.

| Baseline characteristics | | No. | Median, days | HR (95%CI) | *P* |
| --- | --- | --- | --- | --- | --- |
| Gender | Male | 226 | 1528 |  |  |
|  | Female | 265 | 1454 | 0.95(0.71-1.29) | 0.765 |
| Age | <65 | 243 | 1501 |  |  |
|  | ≥65 | 248 | 1268 | 1.27(0.94-1.72) | 0.120 |
| Cancer stage | stage I | 265 | 1492 |  |  |
|  | stage II | 116 | 1209 | 2.36(1.62-3.44) | <0.001 |
|  | stage III | 79 | 807 | 3.55(2.40-5.24) | <0.001 |
|  | stage IV | 24 | 976 | 3.48(1.94-6.24) | <0.001 |
|  | Missing case | 7 |  |  |  |

Note. Seven samples were excluded because of missing value for cancer stage. These tests were two-side.

Abbreviations: HR (95%CI) = Hazard ratios, 95% confidence intervals; *P* = *P* values were estimated from Cox proportional regression models.

Table S2. Primer sequences used in the study.

| **Name** | **Sequence** |
| --- | --- |
| GAPDH -Forward primer | 5′-CAGGAGGCATTGCTGATGAT-3′ |
| GAPDH -Reverse primer | 5′-GAAGGCTGGGGCTCATTT-3′ |
| ITGB8-Forward primer | 5′-TGTGAAGCAGGCAGATGCCAATG-3′ |
| ITGB8-Reverse primer | 5′-GCCTCTTCCACTGCACACTTGG -3′ |
| COMP-Forward primer | 5′-CACAGAGCGTTCCGCAGCTGTTC-3′ |
| COMP-Reverse primer | 5′-AACACGGTCACGGATGACGACTATG-3′ |
| TP53-Forward primer | 5′-TGCGTGTTTGTGCCTGTCCTG-3′ |
| TP53-Reverse primer | 5′-TTGTTGGGCAGTGCTCGCTTAG-3′ |
| EGFR-Forward primer | 5′-GTGTGCCACCTGTGCCATCC-3′ |
| EGFR -Reverse primer | 5′-GCCACCACCAGCAGCAAGAG-3′ |
| EGFR^L858R^-Forward primer | 5′-GCATTTGCCAAGTCCTACAGA-3′ |
| EGFR^L858R^ -Reverse primer | 5′-CTATCAATGCAAGCCACGG-3′ |

[Firgure S1](http://clincancerres.aacrjournals.org/content/23/20/6078.long#T1). Workflow of the whole analysis for Patients in TCGA lung adenocarcinoma cohort.


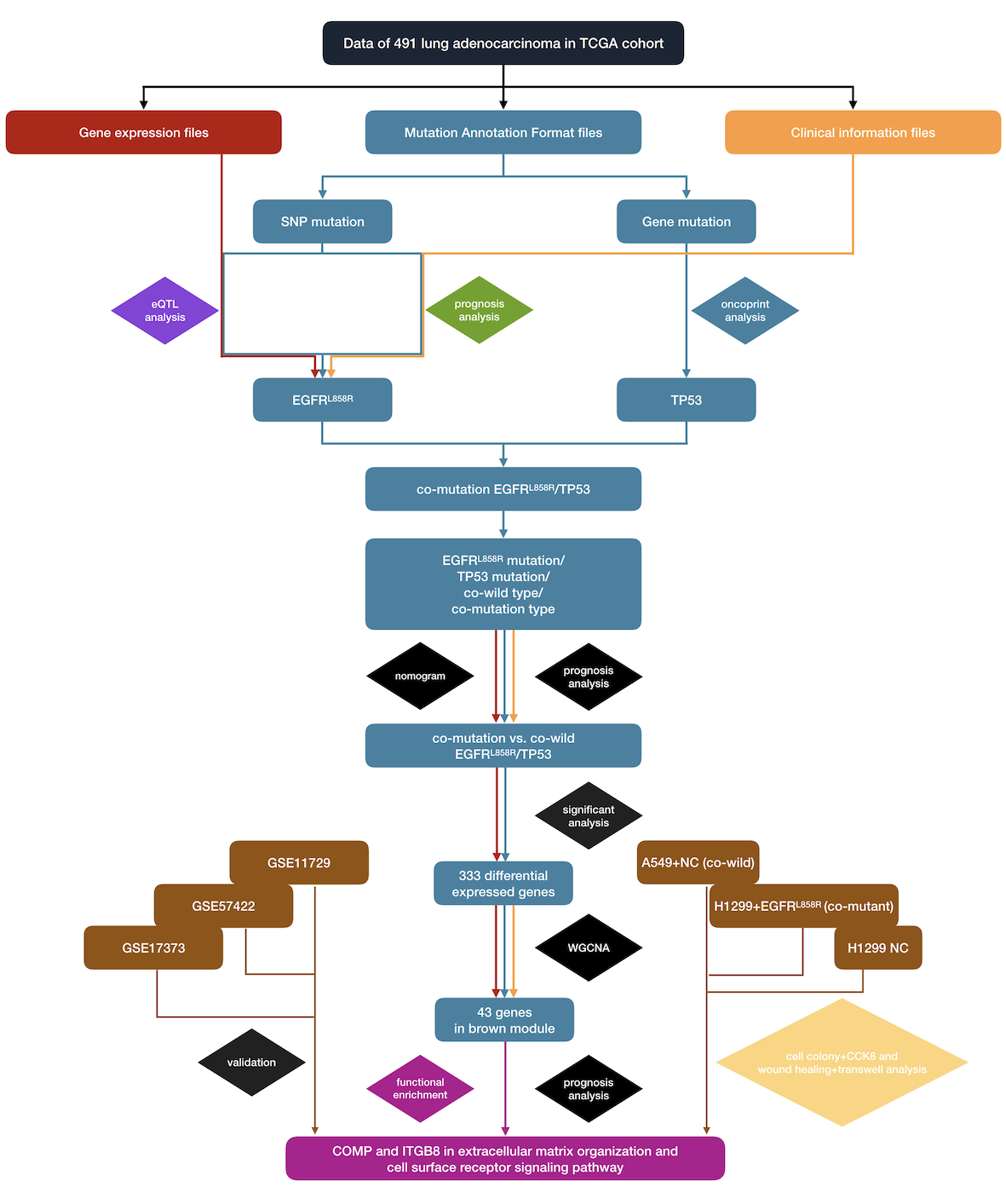

Supplement: Supplementary file 1 [file Table_1.DOCX]
